# Identification of natural product modulators of Merkel cell carcinoma cell growth and survival

**DOI:** 10.1038/s41598-021-93097-9

**Published:** 2021-06-30

**Authors:** Emily A. Smith, Natasha T. Hill, Tara Gelb, Khalid A. Garman, Ekaterina I. Goncharova, Heidi R. Bokesch, Chang-Kwon Kim, Karen L. Wendt, Robert H. Cichewicz, Kirk R. Gustafson, Isaac Brownell, Curtis J. Henrich

**Affiliations:** 1 Molecular Targets Program, National Cancer Institute, Frederick, MD 21702 USA; 2 Basic Science Program, Frederick National Laboratory for Cancer Research, Frederick, MD 21702 USA; 3 Dermatology Branch, National Institute of Arthritis and Musculoskeletal and Skin Diseases, Bethesda, MD 20891 USA; 4 Advanced Biomedical Computational Science, Frederick National Laboratory for Cancer Research, Frederick, MD 21702 USA; 5 Natural Products Discovery Group, Department of Chemistry & Biochemistry, Institute for Natural Products Applications and Research Technologies, University of Oklahoma, Norman, OK 73019 USA

**Keywords:** High-throughput screening, Skin cancer, Fungi, Natural products

## Abstract

Merkel cell carcinoma (MCC) is a rare, but aggressive skin cancer the incidence of which has increased significantly in recent years. The majority of MCCs have incorporated Merkel cell polyomavirus (VP-MCC) while the remainder are virus-negative (VN-MCC). Although a variety of therapeutic options have shown promise in treating MCC, there remains a need for additional therapeutics as well as probes for better understanding MCC. A high-throughput screening campaign was used to assess the ability of > 25,000 synthetic and natural product compounds as well as > 20,000 natural product extracts to affect growth and survival of VN-MCC and VP-MCC cell lines. Sixteen active compounds were identified that have mechanisms of action reported in the literature along with a number of compounds with unknown mechanisms. Screening results with pure compounds suggest a range of potential targets for MCC including DNA damage, inhibition of DNA or protein synthesis, reactive oxygen species, and proteasome inhibition as well as NFκB inhibition while also suggesting the importance of zinc and/or copper binding. Many of the active compounds, particularly some of the natural products, have multiple reported targets suggesting that this strategy might be a particularly fruitful approach. Processing of several active natural product extracts resulted in the identification of additional MCC-active compounds. Based on these results, further investigations focused on natural products sources, particularly of fungal origin, are expected to yield further potentially useful modulators of MCC.

## Introduction

Merkel cell carcinoma (MCC) is a rare and aggressive neuroendocrine carcinoma of the skin^1–3^. MCC has histological properties that led to the initial belief that this cancer started in Merkel cells; however it is currently debated from which cell type this cancer originates^2,3^. MCC incidence has increased substantially in recent years^4,5^. Current treatment of MCC is surgery and radiation for localized tumors and immunotherapy for advanced disease^3,6^. Due to the highly aggressive nature of the tumors, reoccurrence is typically observed^6^. Immunotherapies have shown promise as a monotherapy for MCC^1,6^ but more effective treatments are still needed.

There are two mechanisms leading to MCC carcinogenesis: integration of the Merkel cell polymavirus (MCPyV) into the host genome or accumulation of ultraviolet light (UV) mutations. Clonal integration of MCPyV DNA into the host genome is observed in approximately 80% of MCC tumors^3^. The integrated viral genome invariably has a truncating mutation of the large T antigen (LT) that is hypothesized to be required for MCC pathogenesis^3^. The truncated LT and small T antigen (ST) are viral oncogenes expressed in virus positive MCC (VP-MCC) tumors that are potential therapeutic targets^3^. The remaining 20% of MCC tumors are virus negative (VN-MCC) and are associated with UV induced DNA damage leading to a high mutational burden^3^. Inactivation of tumor suppressor genes and activation of protooncogenes lead to possible treatable targets in major pathways involved in tumorigenesis^1^. Some alterations are shared between both tumor types, while others are only seen in VN-MCC tumors. Although several promising molecular targets have been suggested for therapeutic development^7^, not all aspects of VN-MCC and VP-MCC tumorigenesis are known. Therefore, the discovery of compounds that are selectively cytotoxic could provide potential effective treatments as well as provide information about novel oncodrivers for each tumor type.

A high throughput screen (HTS) was developed to discover possible selective small molecules effective against MCC. In order to simplify and speed development and screening, three cell lines were chosen as representative of VN-MCC (MCC26), VP-MCC (MKL-1) tumor types along with a non-cancerous skin cell line (HaCaT) for determination of specificity. Loss of cell viability was assessed after treatment with 27,753 pure compounds and 20,532 natural product extracts. Active samples from the initial screen were then validated across additional VN-MCC and VP-MCC cell lines. Among the active compounds identified which have known mechanisms of action, many of their targets have been shown to be important in MCC. Compounds and derivatives obtained from natural products comprise a large proportion of approved drugs, particularly for cancer, and continue to provide rich resources for drug discovery^8,9^. Many of the active compounds are natural products and a variety of natural products have also been previously identified as modulators of MCC cell growth and survival^10,11^. To further access the wide chemical diversity extant in natural products, a library consisting of both crude and partially purified natural product extracts from marine, plant, bacterial, and fungal sources was assessed. Identification of natural product extracts that affect MCC cell viability can be expected to lead to discovery of additional new compounds with potentially novel mechanisms of action.

## Materials and methods

### Materials

All cell culture components were from Invitrogen (Carlsbad, CA, USA). Clear 384‐well tissue culture‐treated assay plates were from Perkin‐Elmer (Waltham, MA, USA). 2,3‐bis[2‐methoxy‐4‐nitro‐5‐sulfophenyl]‐2*H*‐tetrazolium‐5‐carboxanilide (XTT) and natural products were obtained from the Drug Synthesis and Chemistry Branch, Developmental Therapeutics Program, Division of Cancer Treatment and Diagnosis, National Cancer Institute (Frederick, MD, USA). Other pure compounds were from commercial or academic chemistry sources. Natural product extracts were obtained from the Natural Products Support Group of the Developmental Therapeutics Program, Division of Cancer Treatment and Diagnosis, National Cancer Institute (Frederick, MD) and the University of Oklahoma Fungal Repository (Norman, OK). LOPAC1280 Library of Pharmacologically Active compounds was from Sigma (St. Louis, MO). Available confirmed active compounds were either obtained from the original supplier or were purchased from Sigma (St. Louis, MO). Chemical structures were rendered in ChemDraw (Perkin-Elmer, Waltham, MA).

### Cell lines used

Three VN-MCC lines (MCC13 and MCC26^12^, and UISO^13^), along with 3 VP-MCC cell lines (MKL-1^14^, MKL-2^15^, and WaGa^16^) as well as HaCaT immortalized keratinocytes^17^ were used.

### Differential growth inhibition/cytotoxicity assay

VN-MCC and VP-MCC cell lines were maintained in RPMI supplemented with 10% fetal bovine serum and penicillin–streptomycin. The control cell line, HaCaT, was maintained in DMEM supplemented with 10% fetal bovine serum and 1% penicillin–streptomycin. Adherent subconfluent cells (HaCaT, MCC13, MCC26, UISO) were harvested by trypsinization and resuspended in the same medium. The non-adherent cell lines (MKL-1, MKL-2, WaGa) were treated with Accutase and passed through a cell strainer to establish a single cell solution suspension. Cells were counted, then transferred to 384‐well tissue culture‐treated plates (45 μL per well) using a μFill microplate liquid dispenser (BioTek, Winooski, VT, USA). Cells were incubated for 4 h before treatment with test or control samples (prediluted to 10 × final concentration in RPMI). For primary screening, cell viability was estimated by XTT assay^18^ after 3 days of incubation. Based on XTT response and optimization, the VN-MCC cell line and the HaCaT cell line were plated at 2500 cells/well while the VP-MCC cell line was plated at 15,000 cells/well for screening. Pure compound libraries were assessed at 1 and 10 μM and natural product extracts (prefractionated and crude) at 1 and 10 µg/ml for their ability to inhibit growth. In total, more than 45,000 samples from a variety of sources were screened. For initial assay development and hit follow-up studies, active growth inhibitory/cytotoxic samples were assessed in a dose–response format using the Promega CellTiter Glo Assay according to the manufacturer’s protocol using 2500 cell/well for all cell lines.

### Choice of cell lines and control compounds for assay development

Several virus negative (VN-MCC) and virus positive (VP-MCC) cell lines have been established and characterized. Among the most widely used VN-MCC cell lines are MCC13, MCC26, and UISO while commonly used VP-MCC cells include MKL-1, MKL-2, and WaGa. These cell lines along with an immortalized human skin cell line (HaCaT) were chosen for preliminary development. For assay development control compounds, navitoclax, pluripotin, and bortezomib were used for assessing differential and general cell killing based on prior experience with these cell lines. Navitoclax (ABT-263) modulates BCL-2 family proteins and was reported to induce cell death in MCC cells^19^. Similarly, bortezomib has been reported to induce cell death in MCC cells at low nanomolar concentrations^20^. An additional control compound, pluripotin was chosen based on reported MCC expression profiles. Pluripotin (SC-1) is an inhibitor of RasGAP, ERK, and S6 kinase^21,22^ and a proteomic study suggested involvement of S6 kinase in MCC^23^.

### Isolation and characterization of natural products

Sources and isolation and identification of natural products from active extracts are described in the Supplementary Information section.

### Data analysis

All XTT‐ and CellTiterGlo-derived cell numbers were normalized to untreated (DMSO only) controls on the same plates. Dose–response curves (during assay development and hit confirmation) were graphed and IC_50_ values estimated using GraphPad Prism 8 (Graphpad Software, San Diego CA, USA, https://www.graphpad.com/scientific-software/prism/) 4-parameter logistic nonlinear regression analysis. ‘Hit’ identification was based on inhibition of MCC cells as discussed in the Results and Discussion sections. For further analysis and comparisons of active compounds, the Log of the IC_50_ was determined in Excel and used to generate a heatmap in R using the gplots, RColorBrewer and colorspace packages.

## Results

### Activity of control compounds against MCC cell lines

**Figure 1:**
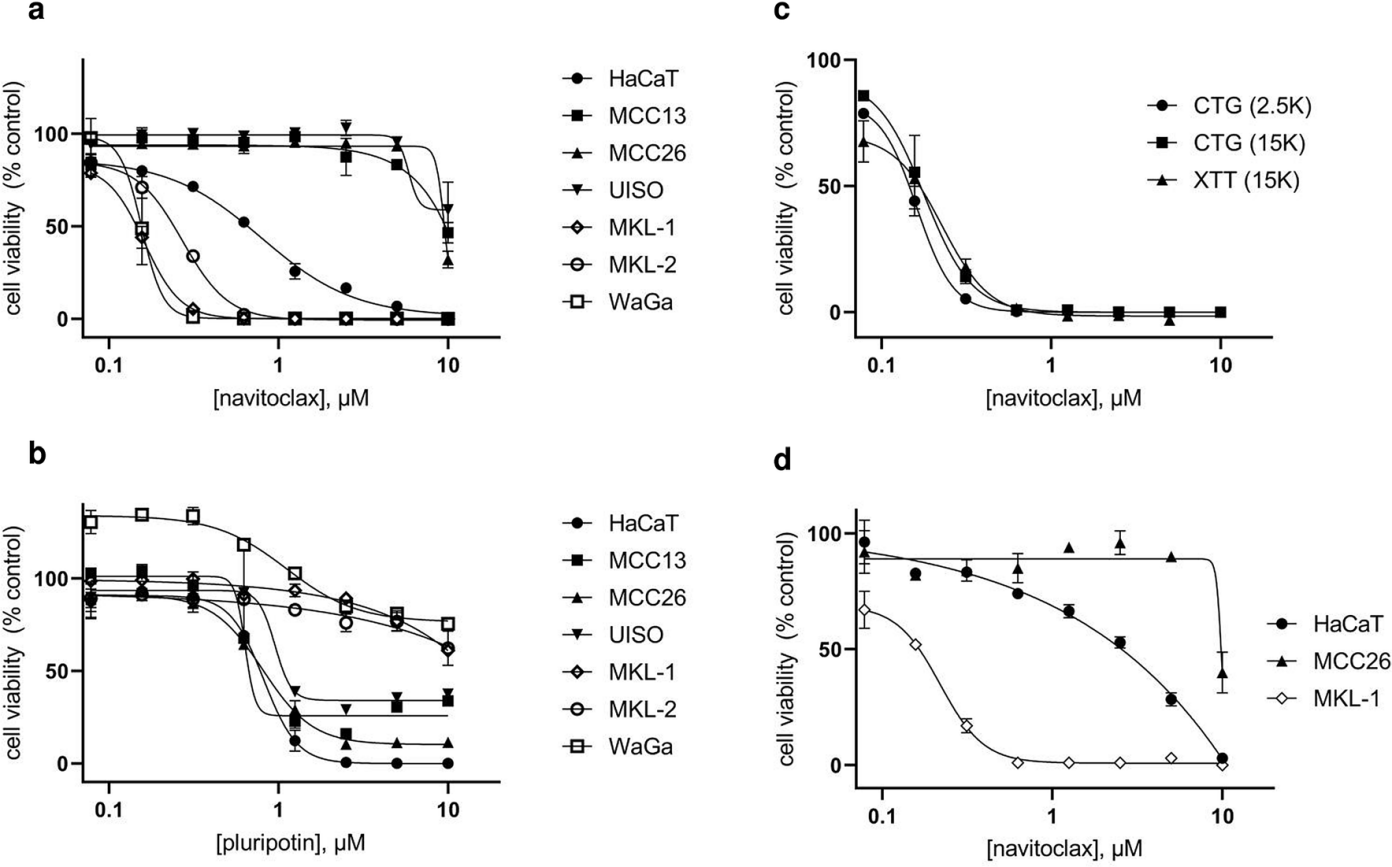
Effects of control compounds on cell survival. The indicated cell lines (2500 cells/well, 384-well plates) were incubated for three days with (**a**) navitoclax (ABT-263), or (**b**) pluripotin (SC-1) and cell viability was estimated using CellTiterGlo (CTG). In a separate experiment, (**c**) MKL-1 cells at 2500 or 15,000 cells/well as indicated were treated with navitoclax and cell viability was estimated with XTT or CTG as indicated. (**d**) XTT assessment of HACAT (2500 cells/well), MCC26 (2500), or MKL-1 (15,000) after navitoclax treatment. In all cases, values were normalized to DMSO controls for each cell line. Error bars represent sd (n = 4).

Navitoclax showed significantly higher potency against VP-MCC cell lines with minimal effects on VN-MCC cells and an intermediate potency against HaCaT cells (Fig. 1A). By contrast, pluripotin preferentially affected VN-MCC and HaCaT cells with little effect on the viability of VP-MCC cells (Fig. 1B). Bortezomib potently eliminated viable cells in all six MCC cell lines and HaCaT—average IC_50_ 11 nM (data not shown). To expedite screening and decrease complexity of data analysis, three cell lines were chosen for development of the primary screen: MCC26, MKL-1, and HaCaT. For ease of comparison of results to previous cytotoxicity/growth inhibition assays in the lab, an XTT-based assay^18^ rather than CellTiterGlo was selected for high-throughput application. A much higher cell number (15,000 cells/well) was required to obtain a reliable XTT signal for MKL-1 cells (data not shown). Navitoclax was therefore used to confirm that the higher cell number was not detrimental to the proposed screening assay configuration. As shown in Fig. 1C, the navitoclax effect on MKL-1 cells was the same whether cell survival was assessed by XTT (at 15,000 cells/well) or CellTiterGlo (at 2500 or 15,000 cells/well). IC_50_ for navitoclax on MKL-1 cells was 200 ± 29 nM (ave ± sd) for the curves shown in Fig. 1. Similarly, XTT analysis of the effects of navitoclax on the other cell lines used in the screening (Fig. 1D) were comparable to those obtained with CellTiterGlo.

### Assay development/optimization/repeatability

Cell densities and incubation times were re-assessed for optimization of the XTT assay for MCC26, MKL-1, and HaCaT cells. Supporting Information Figure S1 shows the effect of variation of both cell numbers and incubation times on XTT signals. For HaCaT and MCC26 cells, 2500 cells/well is within the linear range after 1–3 day incubation and signal to background ratio is maximized at 3 days. For MKL-1, signal at 15,000 cells/well is also in the linear range at 3 days, but maximal at 4 days. However, MKL-1 response to navitoclax treatment was considerably more variable at 4 days (Fig. S1). Based on these results, a 3 day treatment time was chosen for the HTS.

The LOPAC 1280 compound library was used to assess assay repeatability and to determine test concentrations and hit definitions. The assay was run multiple times over several days at 1 and 10 µM. Scatter plots of the average responses of each cell line to each compound are shown in Supporting Information Fig. S2. The coefficient of variation (CV) ranged from 10 to 27% depending on cell line and concentration. The average Z’-factor, a measure of assay quality^24^, for each cell line is above 0.6 indicating a good assay window under the optimized screening conditions. Z’ was calculated for all assay plates to monitor assay performance throughout the screening campaign. Reproducibility for repeats of individual samples and across the plates is detailed in Table 1 for each cell line. Active compounds (*i.e.*, < 50% cell survival at 1 and/or 10 µM) for each cell line were re-assessed in dose–response format for confirmation.

**Table 1:** Assay reproducibility—repeat assays with LOPAC compounds.

Cells	CV (individual compounds)^a^		CV (plate average values)^b^		Average Z′^c^
	1 µM	10 µM	1 µM	10 µM	
HaCaT	10.9	15.4	4.1	4.8	0.71
MCC26	18.5	20.6	10.8	11.3	0.61
MKL-1	17.1	27.0	3.2	13.5	0.60

CV = sd/average × 100%.

^a^average CV for each of 1280 compounds (n = 3 repeats per cell line).

^b^average CV for mean plate response (n = 3 repeats per cell line).

^c^Average Z′, n = 24 plates per cell line (LOPAC only).

### Application to high throughput screening

To maximize the probability of identifying samples with interesting activities against MCC cells, an increasingly stringent sequential assay process was defined. Primary screening was performed by treating MCC26, MKL-1, and HaCaT cells at 1 and 10 µM (or 1 and 10 µg/ml for natural product extracts) for 3 days. Samples that reduced cell survival to < 50% of control for one or both MCC cell lines at either concentration were considered as primary hits. Samples that affected all three cell lines at both concentrations were considered to be generally toxic and not further pursued. Primary hits were then assessed in a dose–response formats, first using XTT in a 5-point 1:2 dilution series (10–20 µM starting concentration for compounds, 10–20 µg/ml for natural product extracts). Agents with an IC_50_ < 10 µM were then tested with a 10-point 1:2 dilution series (starting concentration typically 10 µM or 10–20 µg/ml) using CellTiterGlo. An IC_50_ less than 2 µM (or 10 µg/ml for extracts) in this assay was considered active. Finally, in order to corroborate selectivity, remaining active samples were assessed in 10-point 1:2 dilution series with CellTiterGlo for the seven cell lines discussed above. In the CellTiterGlo experiments, all cells were assessed at 2500 cells/well (see Table 2 for screening statistics). The numbers in the table indicate samples that continued to exhibit potent effects when assessed against all 7 cell lines which comprised the majority of the active samples. However, a few samples (for example, tonantzitlolone and caulibugulone B – see Supporting Information Fig. S3) appeared to have selective activity when three cell lines were assessed, but actually only had activity against a single cell line once all seven were assessed.

**Table 2:** HTS results.

	Pure compounds	Natural product extracts
Total screened	27,753	20,532
Primary hits^a^	1214	279
IC_50_ < 10 µM or 10 µg/ml (XTT)^b^	93	103
IC_50_ < 1–2 µM or 10 µg/ml (CTG)^c^	36	55
7 cell lines^d^	21	45

^a^Viability reduced to < 50% at 3 days in at least one MCC cell line (MCC26 or MKL-1) and no general toxicity using 1 or 10 µM of pure compounds, 1 or 10 µg/ml of natural product extracts.

^b^IC_50_ < 10 µM with 5-point dose response curve.

^c^IC_50_ < 2 µM with 10-point dose response curve.

^d^Tested across 6 MCC cell lines plus HaCaT control cells. For natural product extracts, confirmed active samples were those that maintained a differential effect in each of the sequential steps.

### Active compounds identified by high-throughput screening

**Figure 2:**
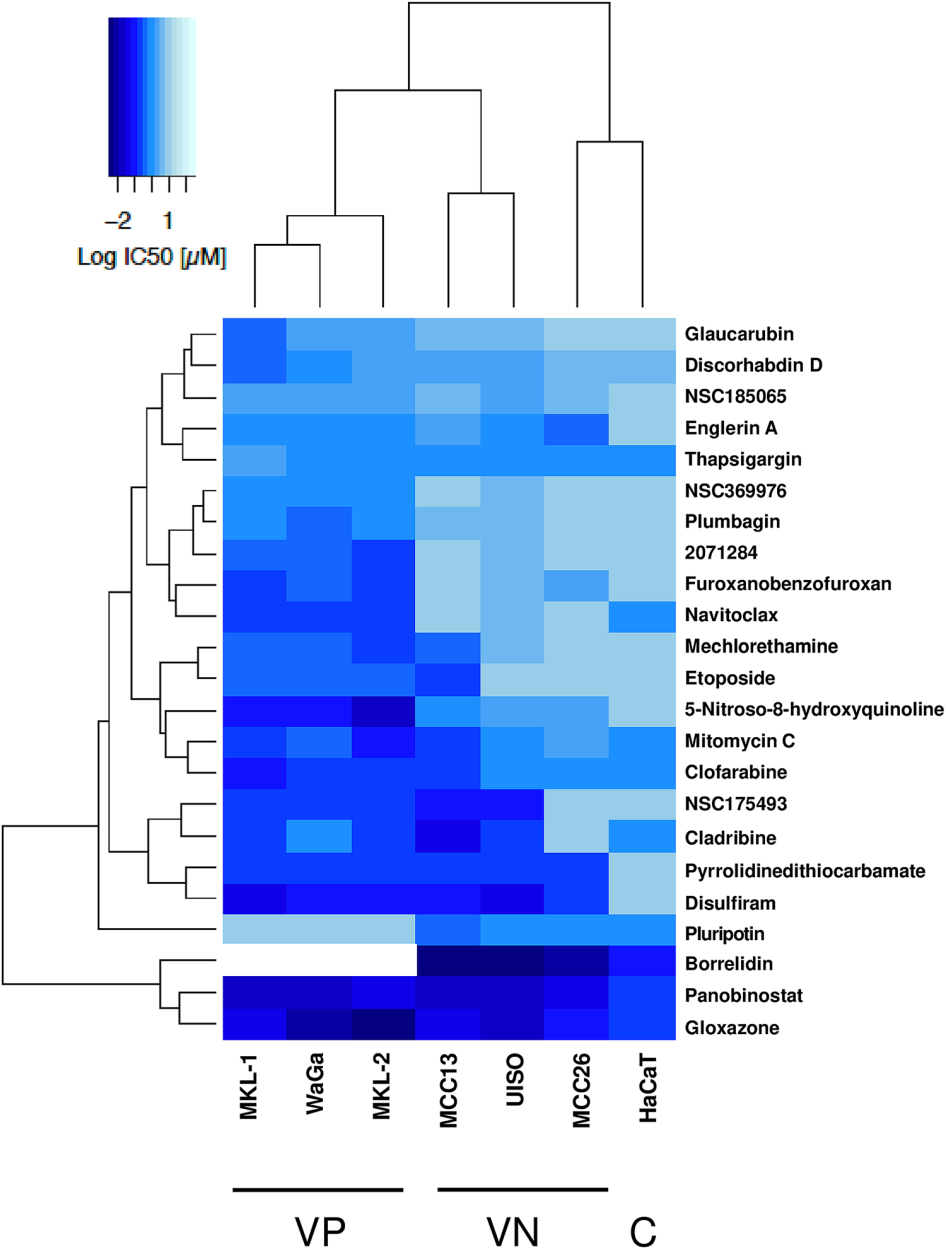
Visualization of drug effectiveness in MCC and control cell lines. VP-MCC, VN-MCC and HaCaT (control) cells were treated with 23 compounds for 72 h. CellTiter Glo assay was used to measure cell viability and determine the IC_50_ for each compound. The log of the IC_50_ was determined and visualized in a heatmap generated in R version 1.3.1073 (R Core Team (2013). R: A language and environment for statistical computing. R Foundation for Statistical Computing, Vienna, Austria. http://www.R-project.org/). Dendrograms indicate cell line and compound similarities based on unsupervised hierarchical clustering. Darker shades indicate higher potency. For borrelidin, IC_50_ could not be calculated (white region) for VP-MCC lines (see Supplementary Figure S4 for dose–response curves for all compounds and cell lines).

A total of 21 known compounds were available from commercial sources or from the National Cancer Institute Developmental Therapeutics Program and showed significant differential activities across seven cell lines. Figure 2 gives a visual representation of the potency [log(IC_50_)] against the 7 cell lines tested of each of the active compounds from the screen along with navitoclax and pluripotin. Complete dose–response curves and structures are provided in Supporting Information (Fig. S4). The majority of the hit compounds had selectivity for VP-MCC cells which was defined as having an average IC_50_ < 50% of the VN-MCC average IC_50_. Two compounds, cladribine and NSC175493 were inactive against MCC26, but active against all of the other MCC lines. Etoposide potently reduced viability of MCC13, but not the other VN-MCC cells and was active against all of the VP-MCC cell lines. Borrelidin appeared to have some specificity for VN-MCC cells but IC_50_ values could not be calculated for VP-MCC cells (see Fig. S4). Cladribine, disulfiram, englerin A, gloxazone, NSC185065, and pyrrolidinedithiocarbamate were similarly effective for VN-MCC and VP-MCC cell lines. With two exceptions (clofarabine and thapsigargin), all of the compounds had IC_50_ values that were on average fivefold lower against VN-MCC and/or VP-MCC as compared to HaCaT cells.

### Natural product extracts and derived compounds

**Figure 3:**
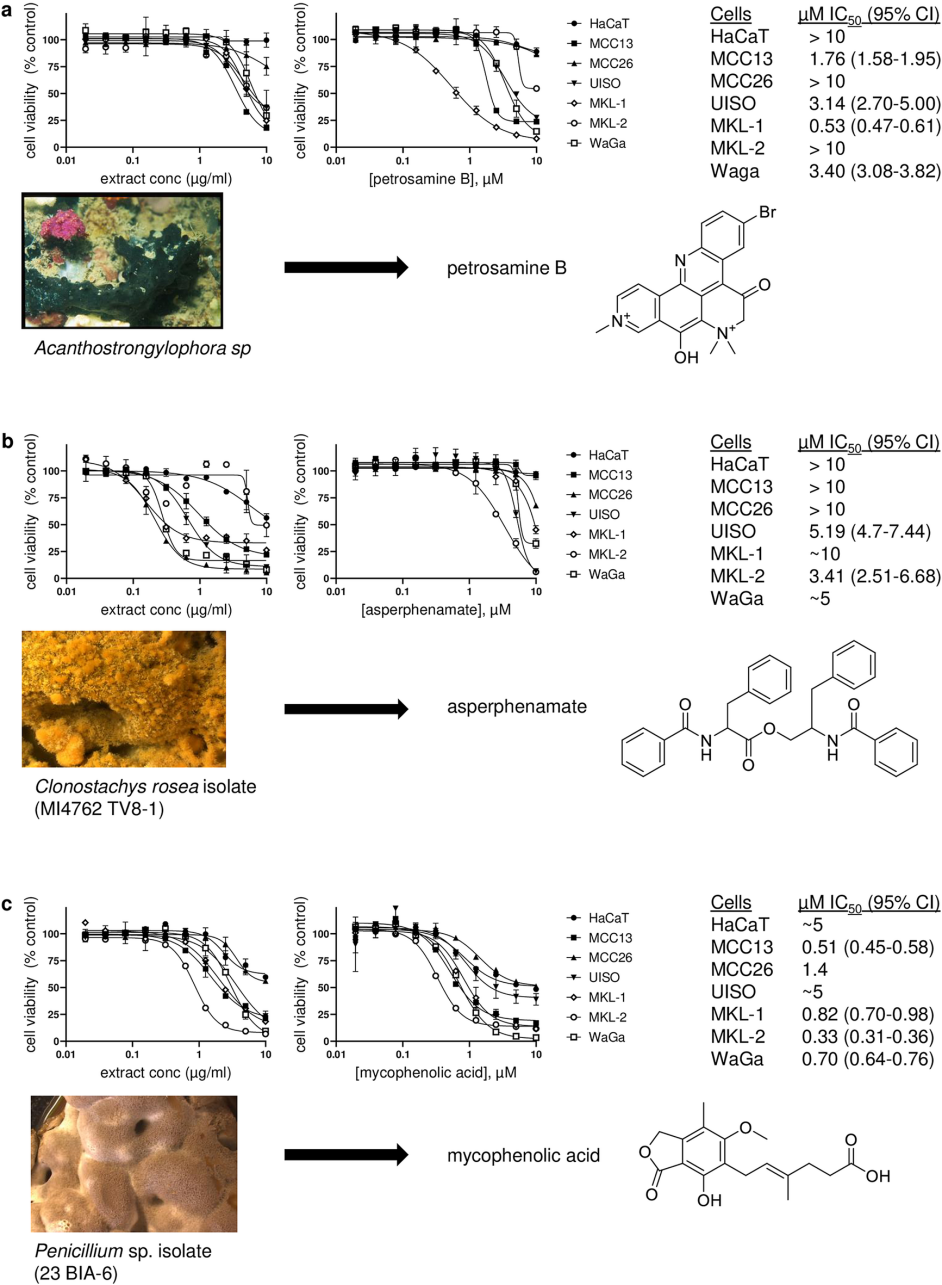
Natural product extracts and derived active compounds. Natural product extracts that reduced MCC cell viability were assessed for activity against the 7 cell lines discussed in the text (left graph in each panel). Pure compounds obtained from these extracts were similarly assessed for activity and IC_50_ values calculated via GraphPad Prism software (right graph and table in each panel). Source organisms and compound isolation and structure elucidation are further discussed in Supplementary Information. Photograph of *Acanthostrongylophora* sp. taken by the Coral Reef Research Foundation under contract to (and provided by) the Natural Products Branch of the National Cancer Institute. Photos of the fungi (*Clonostachys rosea* and *Penicillium* sp.) were obtained the University of Oklahoma. Compound structures were obtained from the PubChem database.

As seen in Table 2, 45 natural product extracts were active and maintained differential activity when assessed against the 7-cell line panel. These extracts consist of potentially complex mixtures of metabolites from each source organisms. Three active natural product extracts were further assessed. In each case, the extracts had significant effects on multiple MCC cell lines and minimal effects on the HaCaT control cell line. As described in Supplementary Information, these extracts were subjected to assay-guided fractionation to purify and identify active components. Processing of each extract yielded a pure compound with differential activity in the 7-cell line panel. Figure 3 shows these results, including the identity of the extracts and structures of the natural products (petrosamine B, asperphenamate, and mycophenolic acid). These compounds were not represented in the pure compound HTS screening libraries.

## Discussion

A multi-tiered high-throughput viability screen successfully identified substances able to selectively target Merkel cell carcinoma cells in general or based on virus status. Many agents that inhibited MCC cell viability also affected control cells with most demonstrating toxicity against all three cell lines at the two doses tested in the initial screen. Samples able to affect one or two of the MCC screening cell lines were reassayed in dose–response format with an orthogonal cell viability assay followed by assessment of activity against two additional VN-MCC and two additional VP-MCC cell lines. Reliance of the screening assay on three cell lines sometimes led to samples that specifically affected only one of the screening lines without subsequent effects on the other MCC cells in the same class (see Supplementary Fig. S3). VN-MCC tumors are reported to be more heterogeneous and have higher mutational burdens than VP-MCC tumors^3^. It is therefore not surprising that the screen identified significantly more compounds with variable effects on VN-MCC cell lines than on VP-MCC cells (Fig. 2 and Fig. S3). In particular, there were VN-MCC outlier cell line responses to cladribine, clofarabine, englerin A, furanobenzofuroxan, mechlorethamine, mitomycin C, and NSC175493.

The majority of compounds identified were selective for VP-MCC cells with only a few showing apparent VN-MCC specificity. Among the 21 active pure compounds identified, 16 have previously been reported to modulate specific molecular targets and/or operate via specific mechanisms of action (in addition, the compound designated 2071284 is an analog of furoxanobenzofuraxan and presumably has a similar MOA). Table 3 lists these compounds sorted by reported mechanism and/or molecular targets. Two of these compounds have been used with MCC in pre-clinical or clinical applications, albeit in combination therapy (etoposide^5^) or for sensitization to immunotherapy (panobinostat^25^). Panobinostat’s efficacy in immunotherapy for MCC is apparently due to it activity as an HDAC inhibitor^25,26^. Another compound with reported HDAC inhibitory activity, 5-nitroso-8-quinolinol was also found in this screen. Although these compounds also have other activities, their presence among the MCC-active compounds identified suggests that HDAC inhibition may not only augment immunotherapy, but may also directly affect the cells. Several compounds including dischorhabdin D, NSC175493, NSC185065, and NSC369976 either apparently are absent from the literature or are simply noted as growth inhibitory/cytotoxic against some cell lines, although NSC175493 may induce DNA damage^27^.

**Table 3:** Active compounds with putative known targets or mechanisms of action.

Compound (NSC#)	Selectivity^a^	Target/MOA	References
5-Nitroso-8-quinolinol (3852)	VP	HDAC inhib/ROS active	^41^
Panobinostat (761190)	VP	HDAC inhib/ROS active	^46^
Plumbagin (688284)	VP	ROS/redox-active, proteasome	^47,48^
Mechlorethamine (762)	VP	DNA damage	^49^
Mitomycin C (26980)	VP	DNA damage	^50^
Etoposide (141540)	VP	topo II/DNA damage	^51^
Gloxazone (82116)	Non	DNA synthesis inhib	^52^
Cladribine (105014)	Non	DNA synthesis inhib	^53^
Clofarabine (606869)	Non	DNA synthesis inhib	^54^
Glaucarubin (14975)	VP	protein synthesis inhib	^55^
Borrelidin (216128)	Uncertain^b^	ptn synth/tRNA synthesis inhib	^56^
Disulfiram (756748)	Non	NFκB, ROS, proteasome	^28,38^
Pyrrolidine dithiocarbamate (298194)	Non	NFκB, ROS, proteasome	^29,31^
Furoxanobenzofuroxan (228139)	VP	Monoamine oxidase inhib	^57^
Thapsigargin (299933)	Non?^c^	Ca^2+^/ER stress	^58^
Englerin A (141540)	Non	PKC/Ca^2+^	^59^

^a^Based on average IC_50_ for VP and VN cell lines.

^b^IC_50_ could not be calculated for VP cell lines.

^c^Incomplete loss of cell viability at highest concentrations.

A number of potential MCC targets and pathways including PI3K/mTOR/AKT, receptor tyrosine kinases and downstream signaling, DNA repair, survivin, BCL-2 family, HDACs, heat shock proteins, MDM2, etc. have been suggested in the literature (see^1,3^ for recent reviews). Many of the compounds identified in this screen have been reported to directly or indirectly affect one or more of these pathways. As seen in Table 3, MCC-selective cell killing can be induced by agents reported to affect a variety of cellular mechanisms, including generation of reactive oxygen species (ROS) and redox effects, effects on DNA synthesis, damage, and repair, inhibition of HDACs, inhibition of protein synthesis, etc. Disulfiram and pyrrolidinedithiocarbamate (PDTC), both NFκB inhibitors^28,29^, are among the most potent compounds identified. However, it has also been shown that disulfiram can affect other cellular processes including proteasomal degradation of proteins and ROS generation^30^. Similarly, PDTC is generally identified as an NFκB inhibitor, but has also been reported to affect proteasomal activity and, depending on conditions, act as a pro- or anti-oxidant^31,32^. MCV polyoma virus small T antigen has variously been reported to *inhibit*^33^ or *activate*^34^ canonical NFκB or to activate non-canonical NFκB^35^. Effects of both PDTC and disulfiram were equipotent against VP-MCC and VN-MCC cells and may be due to their other targets. Five compounds were found to be highly VP-MCC-selective (> fivefold based on average IC_50_ values) with no effect on HaCaT control cells (up to 10 µM) and without significant outlier cell line effects. All five also had sub µM potency against VP-MCC cell lines. These were furoxanobenzofuroxan and its analog 2071284, 5-nitroso-8-hydroxyquinoline, plumbagin, and NSC369976. The only target identified for furoxanobenzofuroxan (and by extension its analog) is monoamine oxidase. Although no clear connection to MCC appears in the literature, monoamine oxidase may be highly expressed in Merkel cells^36^. As seen in Table 3, other targets of active compounds include ROS, HDACs, DNA and protein synthesis, and DNA damage. Although generally considered to be a protein synthesis inhibitor, glaucarubin was reported to induce DNA damage leading to its VP-MCC-selective effects in a previous screen^10^. There were several compounds for which no cellular mechanisms or molecular targets could be found in the literature and a number of those with putative mechanisms have apparently not been extensively studied, in some cases for decades.

Zinc and/or copper binding have been implicated in the activities of several of the compounds identified in this study. These include pyrrolidine dithiocarbamate^31,37^, disulfiram^38^, clofarabine and cladribine^39^, gloxazone [(3-ethoxy-2-oxobutyraldehyde bis(thiosemicarbazone)]^40^, and 5-nitroso-8-quinolinol^41^ suggesting additional potential pathways by which MCC may be attacked. Interestingly, these clustered in the lower half of the heat map shown in Fig. 2.

The plethora of possible mechanisms of action represented by the compounds identified in this screening campaign suggests a potentially wide variety of mechanisms by which MCC cells can be targeted. Many of these compounds, in fact, have multiple reported mechanisms and may require effects on multiple cellular processes to modulate MCC cell growth and survival. Similarly, there was no clear correlation between structural features of active compounds and their potency or selectivity. It is of particular interest that a significant number of the pure compounds identified in the screen are natural products. In fact, several natural products have been reported to have potentially useful activity against MCC cells^10,11^. As a result, a small library of crude or partially purified natural product extracts was also screened yielding active extracts from 45 organisms. These are undergoing assay-guided fractionation to purify and characterize potentially novel modulators of MCC cell growth. Three derived compounds are described here. Mycophenolic acid is an extensively characterized drug with a range of cancer-relevant molecular targets and mechanisms^42^ and asperphenamate is reported to be a cathepsin inhibitor^43^. By contrast, petrosamine B has not been widely studied although it has been reported to inhibit an enzyme from *H. pylori*^44^. The results of the pure compound screen demonstrate that HTS assay was effective in identifying active small molecules with effects consistent with their known MCC-relevant mechanisms of action and/or molecular targets, including some of which have previously shown promise as MCC-targeted agents. Further analysis of new natural products and compounds without known mechanisms of action may reveal additional possible avenues for targeting MCC cells. A range of targets have been reported for several of the individual compounds identified in this study and polypharmacology is often a feature of natural products^45^. Thus, further investigation of natural products may be a particularly fruitful approach for the identification of novel therapeutics and probes. Interestingly, like the sources of mycophenolic acid and asperphenamate, the majority of the MCC-active natural product extracts are from fungal samples sourced via the Citizen Science Soil Collection Program at the University of Oklahoma^60^. From the discovery of penicillin onward, fungi have been rich sources of novel biologically active natural products^61^.

## Supplementary Information

Supplementary Information.
