# Supplementary material for: Identification of natural product modulators of Merkel cell carcinoma cell growth and survival
Source: Sci Rep. 2021 Jun 30;11:13597. doi: 10.1038/s41598-021-93097-9 (PMC8245553; doi:10.1038/s41598-021-93097-9)
Supplement: Supplementary file 1 — Supplementary Information. [file 41598_2021_93097_MOESM1_ESM.pdf]

SUPPLEMENTARY INFORMATION

Identification of Natural Product Modulators of Merkel Cell Carcinoma Cell Growth and Survival

Emily A. Smith,[1,2] Natasha T. Hill,[3] Tara Gelb,[3] Khalid A. Garman,[3] Ekaterina I. Goncharova,[1,4] Heidi R. Bokesch,[1,2] Chang-Kwon Kim,[1] Karen L. Wendt,[5] Robert H. Cichewicz,[5] Kirk R. Gustafson,[1] Isaac Brownell,[3] Curtis J. Henrich*[1,2]

[1]Molecular Targets Program, National Cancer Institute, Frederick, Maryland 21702

[2]Basic Science Program, Frederick National Laboratory for Cancer Research, Frederick, Maryland 21702

[3]Dermatology Branch, National Institute of Arthritis and Musculoskeletal and Skin Diseases, Bethesda, Maryland 20891

[4]Advanced Biomedical Computational Science, Frederick National Laboratory for Cancer Research, Frederick, MD 21702

[5]Natural Products Discovery Group, Institute for Natural Products Applications and Research Technologies, Department of Chemistry & Biochemistry, University of Oklahoma, Norman, OK 73019

CONTENTS

Page 2: Figure S1: *Effects of cell number and incubation time on signal.*

Page 4: Figure S2: *LOPAC scatter plots.*

Page 7: Figure S3: *False positive results due to outlier activity.*

Page 8: Figure S4: *Active compound structures and activities against seven cell lines.*

Page 15: *Natural product source organisms, active compound isolation and structure elucidation.*

Figure S1: *Effects of cell number and incubation time on signal.*

HaCaT, MC26, and MKL-1 cells were plated at the indicated cell densities in 384-well plates followed by assessment of viable cell numbers by XTT after 1-4 d (panels A-D). Background absorbance (450 nm – no cells) averaged 0.16 ± 0.03 with no variation based on culture times. In a separate experiment (panels E and F), MKL-1 cells were plated at the indicated densities followed by addition of navitoclax and XTT assessment after 3 d (E) or 4 d (F). Signal was normalized to untreated (DMSO) control cells in the same plate. Error bars represent sd (n = 4).

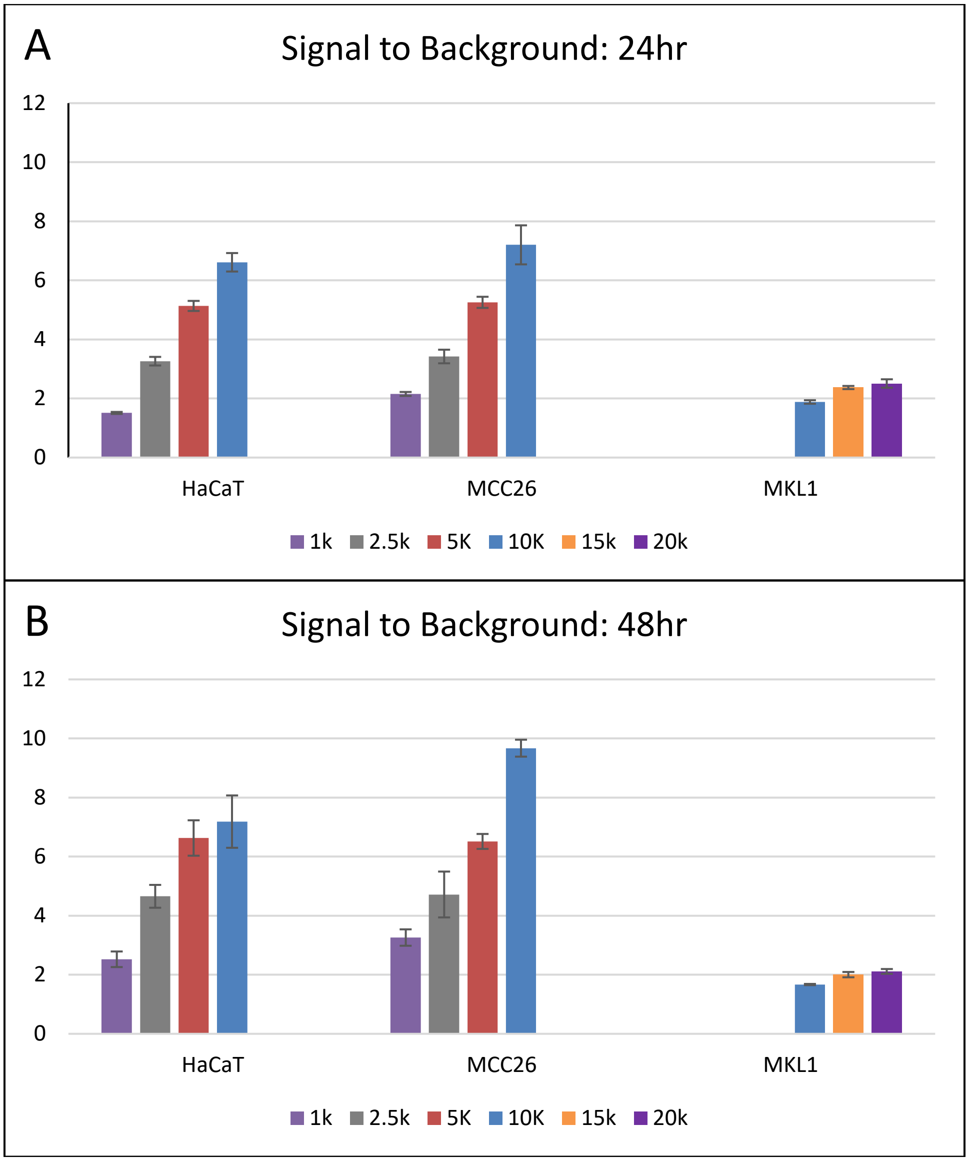

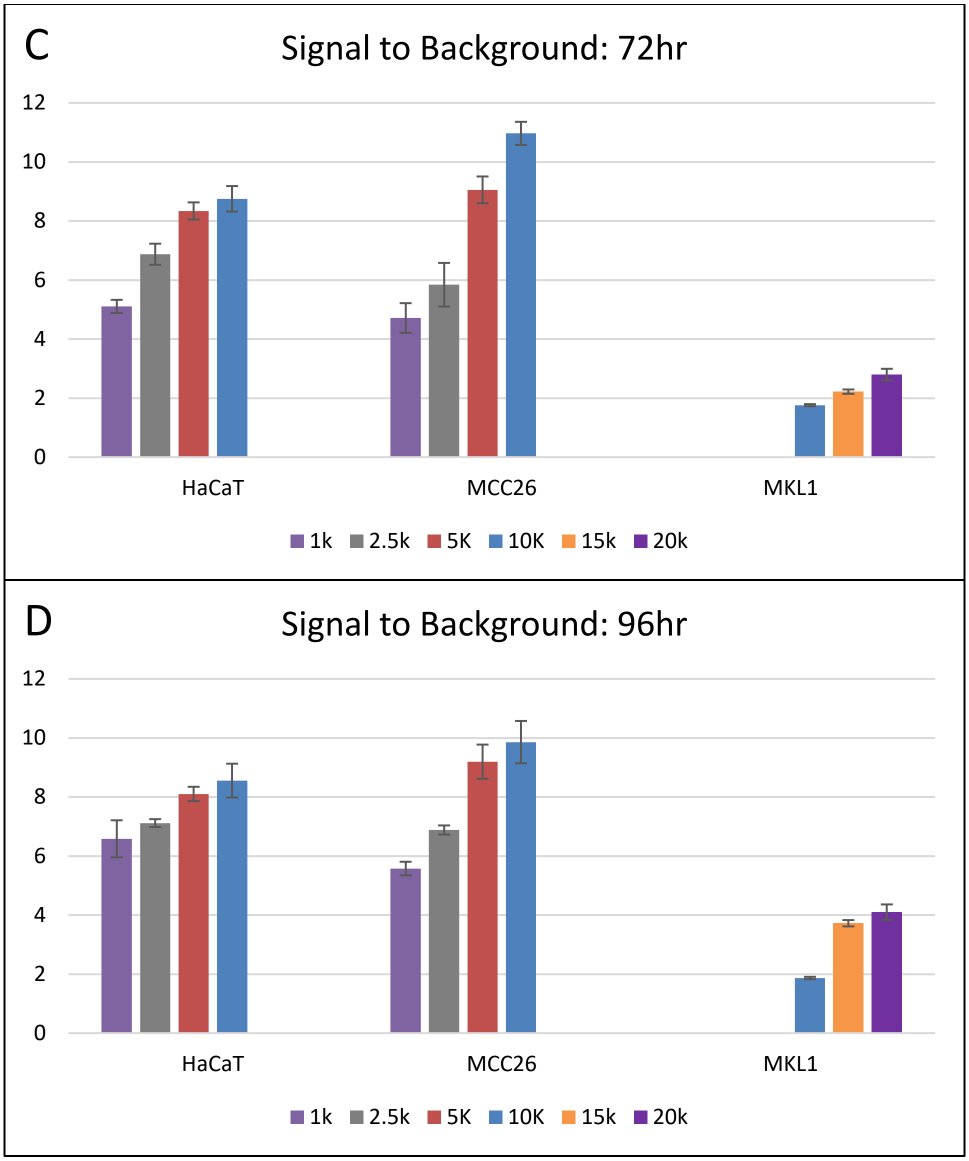
C
Signal to Background: 72hr
12
10
8
6
4
2
0
HaCaT
MCC26
MKL1
1k 2.5k 5K 10K 15k 20k
D
Signal to Background: 96hr
12
10
8
6
4
2
0
HaCaT
MCC26
MKL1
1k 2.5k 5K 10K 15k 20k

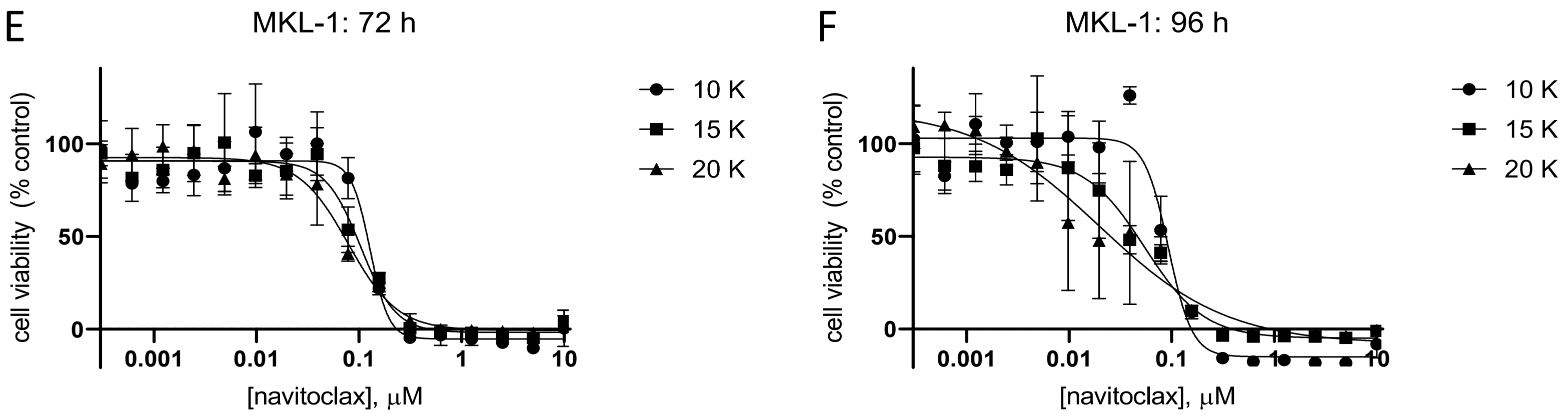
E
MKL-1: 72 h
cell viability (% control)
100
50
0
0.001
0.01
0.1
1
10
[navitoclax], μM
10 K
15 K
20 K
F
MKL-1: 96 h
cell viability (% control)
100
50
0
0.001
0.01
0.1
1
10
[navitoclax], μM
10 K
15 K
20 K

Figure S2: *LOPAC scatter plots*.

The indicated cells were plated at 2500 (HaCaT and MCC26) or 15,000 (MKL-1) cells/well and treated for 3 d with 1 μM (top row in each group) or 10 μM (bottom row) LOPAC compounds followed by assessment of surviving cell numbers by XTT (normalized to untreated (DMSO) control cells in the same plate.

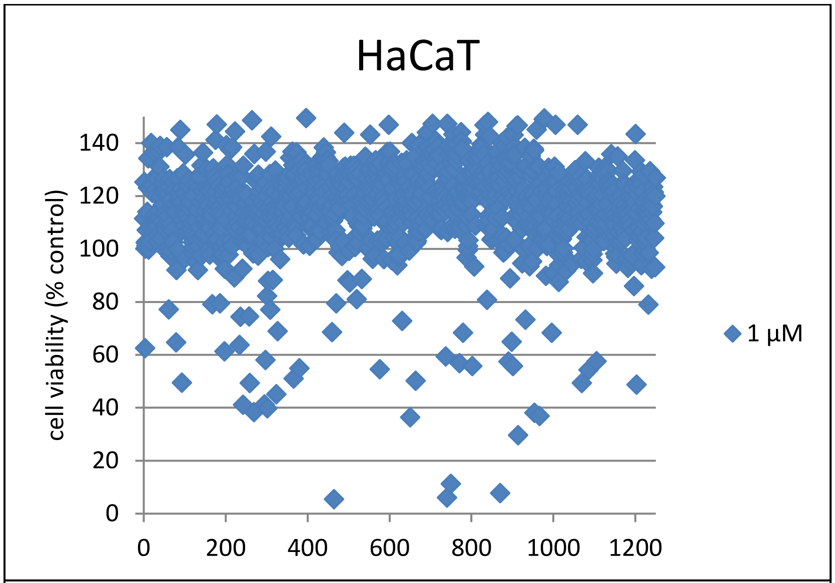

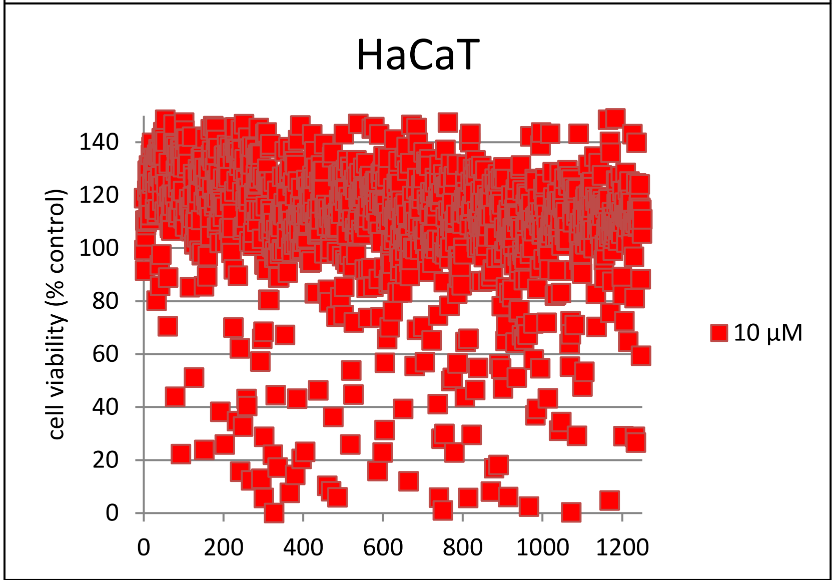

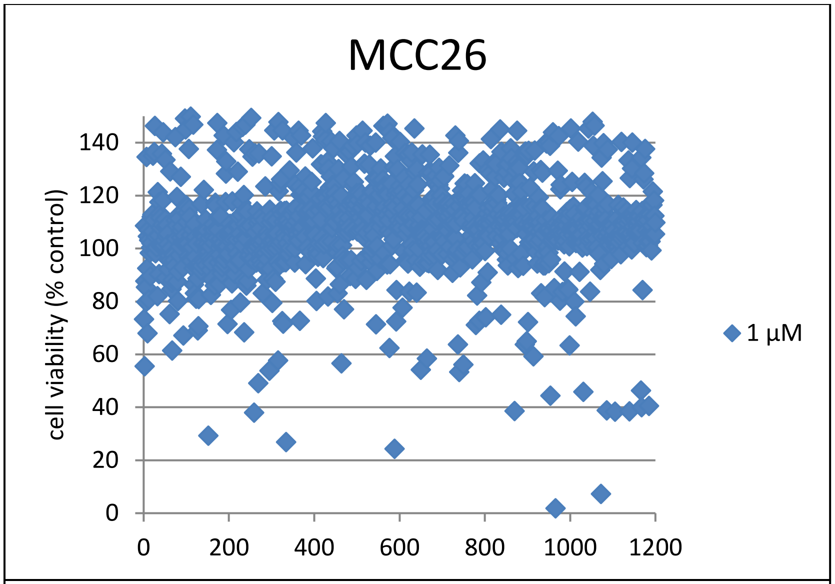
MCC26
cell viability (% control)
140
120
100
80
60
40
20
0
0
200
400
600
800
1000
1200
1 µM

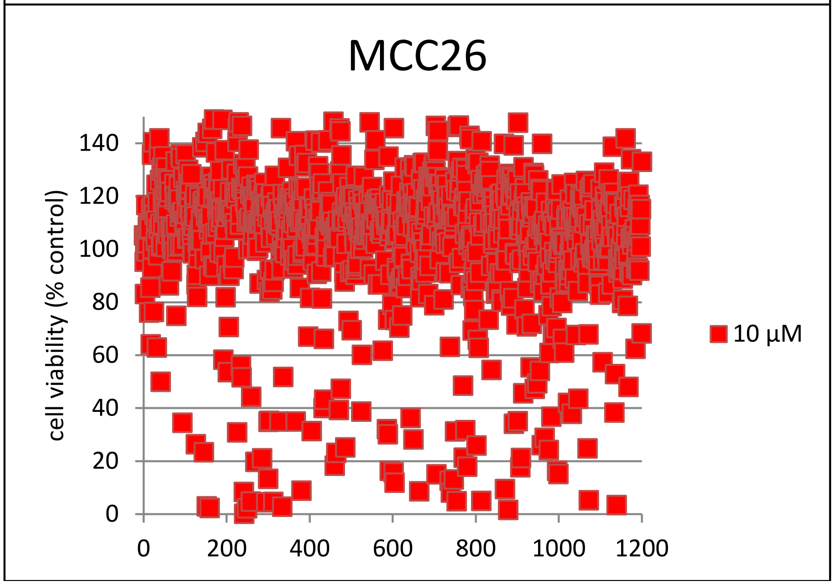
MCC26
cell viability (% control)
140
120
100
80
60
40
20
0
0
200
400
600
800
1000
1200
10 µM

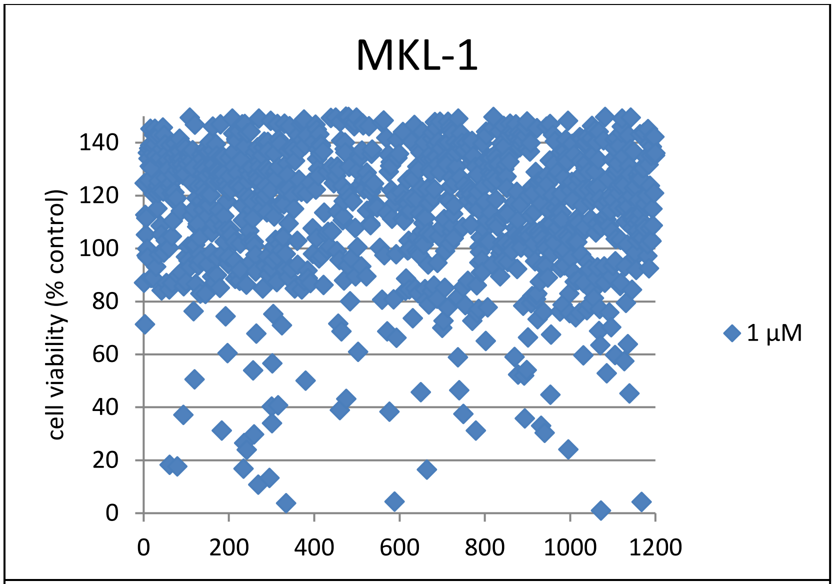
MKL-1
cell viability (% control)
0
20
40
60
80
100
120
140
0
200
400
600
800
1000
1200
1 µM

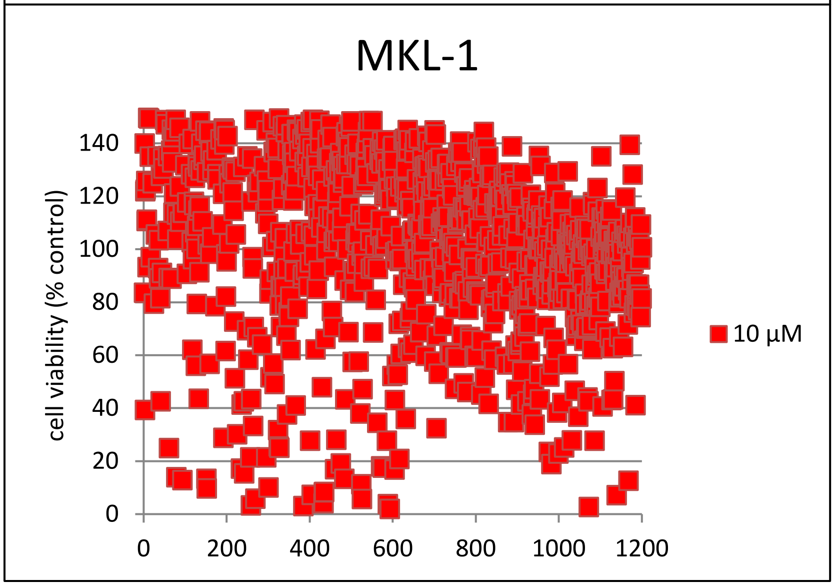
MKL-1
cell viability (% control)
0
20
40
60
80
100
120
140
0
200
400
600
800
1000
1200
10 µM

Figure S3: *False positive results due to outlier activity.*

The indicated cell lines (2500 cells/well, 384-well plates) were incubated for three days with tonantzitlolone (NSC688224 – panels A and B) or caulibugulone B (panels C and D). Surviving cell numbers were estimated using CellTiterGlo and normalized to DMSO controls for each cell line. Error bars represent range (duplicate plates, duplicate wells per plate, panels A and C) or sd (n = 3 plates, duplicate wells per plate, panels B and D).

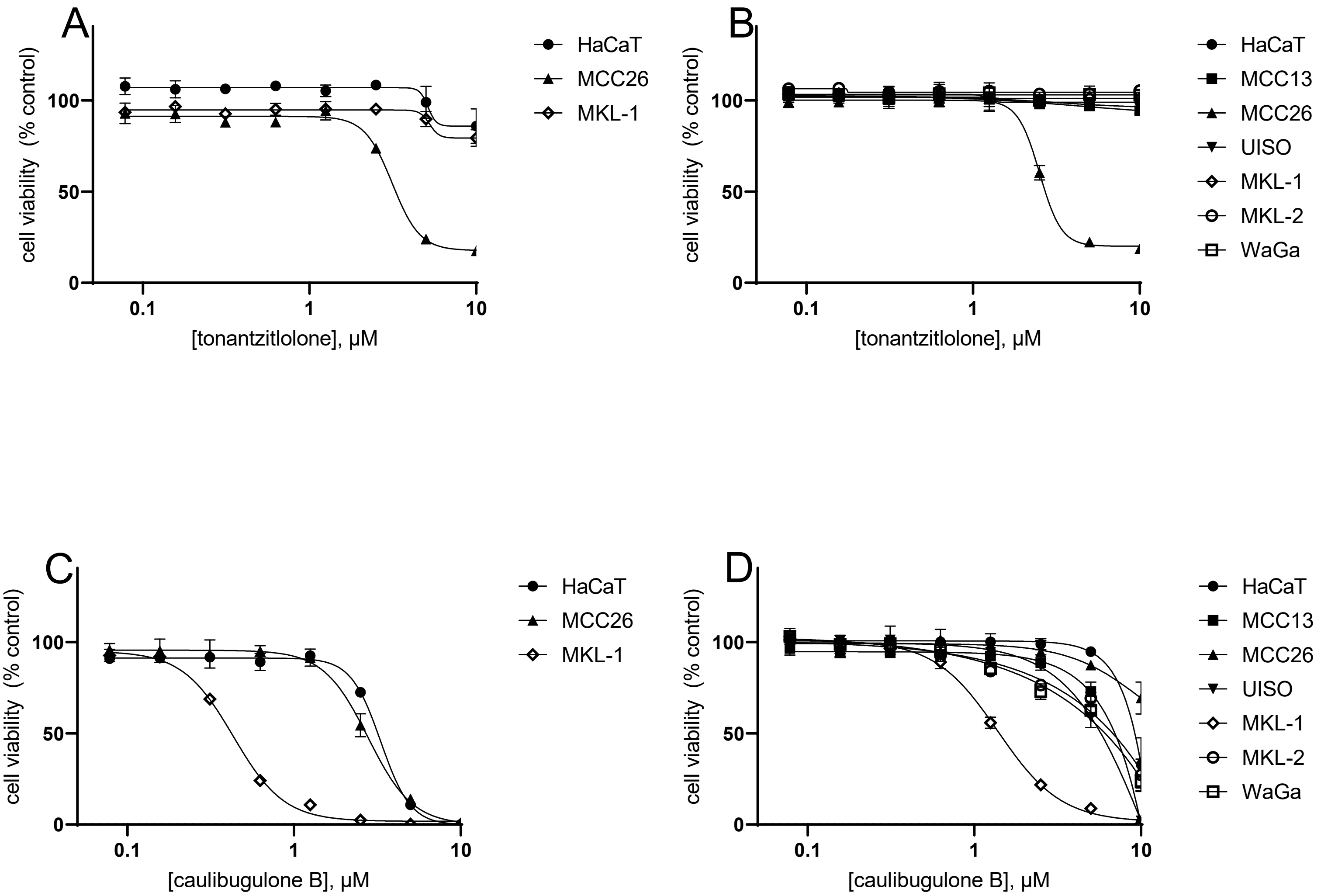

Figure S4: *Active compound structures and activities against seven cell lines.*

The indicated cells were plated at 2500 cells/well and treated with compounds and cell survival assessed by CellTiterGlo (normalized to untreated (DMSO)) cells on the same plate. Structures were obtained from the supplier and/or the PubChem database. Compounds identified in HTS are in the same order as in Figure 2.

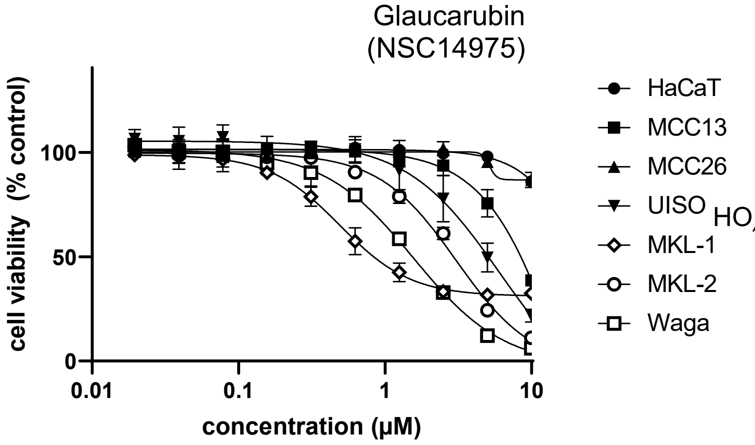

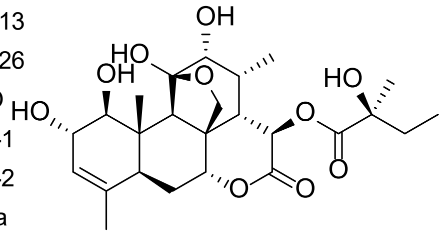

Cell line	μM IC_{50} (95% CI)
HaCaT	> 10
MCC13	~10
MCC26	> 10
UISO	~6
MKL-1	0.49 (0.43-0.57)
MKL-2	3.06 (2.59-4.00)
WaGa	1.56 (1.36-1.86)

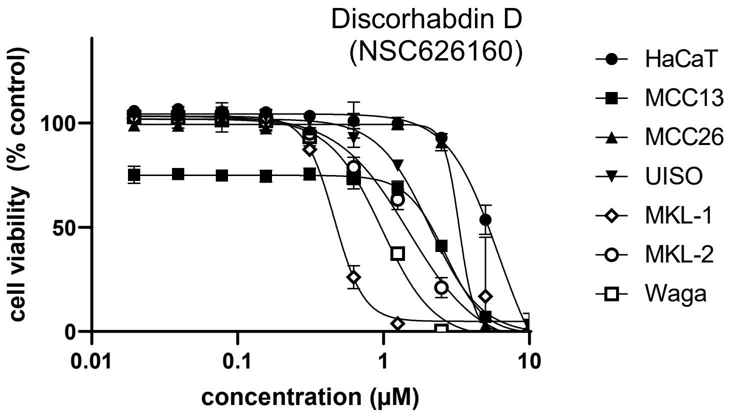

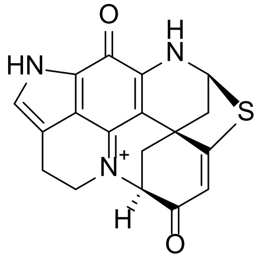

Cell line	μM IC_{50} (95% CI)
HaCaT	6.17 (5.33-8.73)
MCC13	2.67 (2.53-2.83)
MCC26	3.31 (< 3.62
UISO	2.07 (1.93-2.23)
MKL-1	0.46 (0.40-0.53)
MKL-2	1.49 (1.32-1.69)
WaGa	0.97 (0.90-1.04)

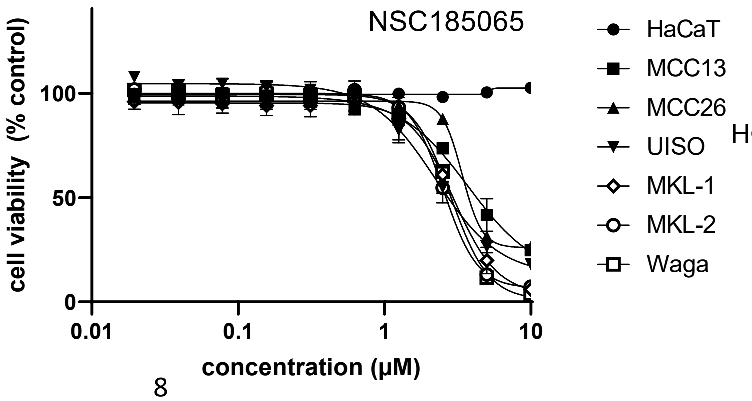

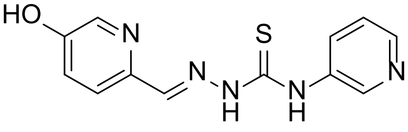

Cell line	μM IC_{50} (95% CI)
HaCaT	> 10
MCC13	3.73 (3.07-5.53)
MCC26	3.40 (3.15-3.60)
UISO	2.24 (2.04-2.50)
MKL-1	2.97 (2.66-3.38)
MKL-2	2.54 (2.39-2.70)
WaGa	2.84 (2.65-3.08)

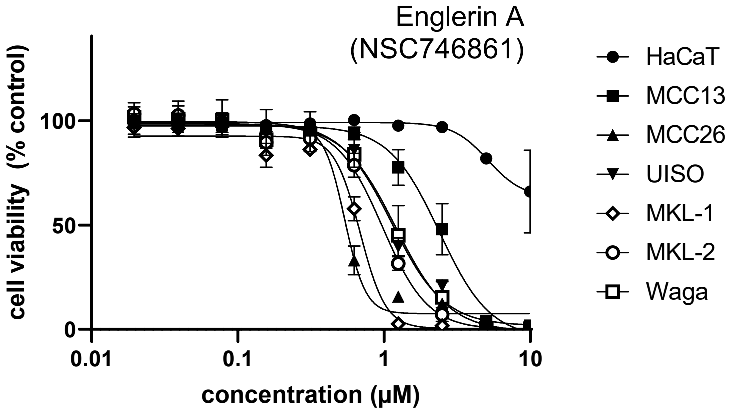

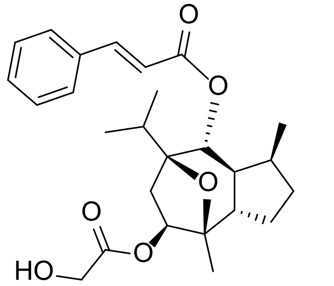

Cell line	µM IC_{50} (95% CI)
HaCaT	~5
MCC13	2.39 (2.14-2.70)
MCC26	~0.5
UISO	1.14 (0.97-1.35)
MKL-1	0.68 (< 0.73)
MKL-2	0.97 (0.89-1.07)
WaGa	1.21 (1.07-1.36)

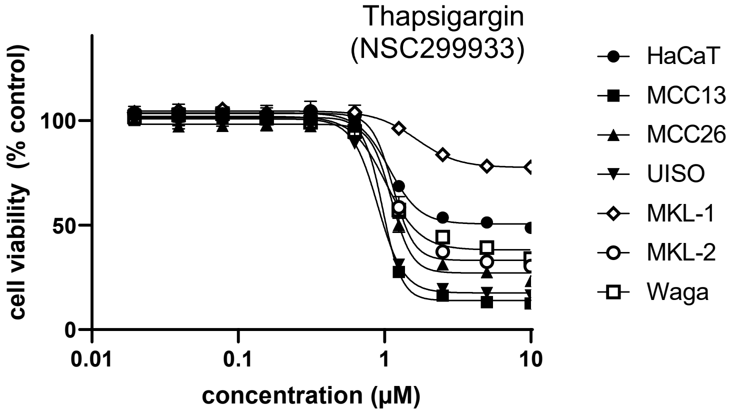

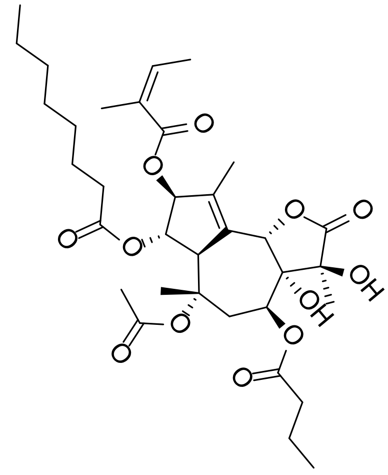

Cell line	µM IC_{50} (95% CI)
HaCaT	1.07 (1.02-1.13)
MCC13	0.97 (0.93-1.03)
MCC26	1.11 (> 1.05)
UISO	0.90 (0.86-0.93)
MKL-1	> 10
MKL-2	1.14 (> 1.08)
WaGa	1.02 (0.94-1.11)

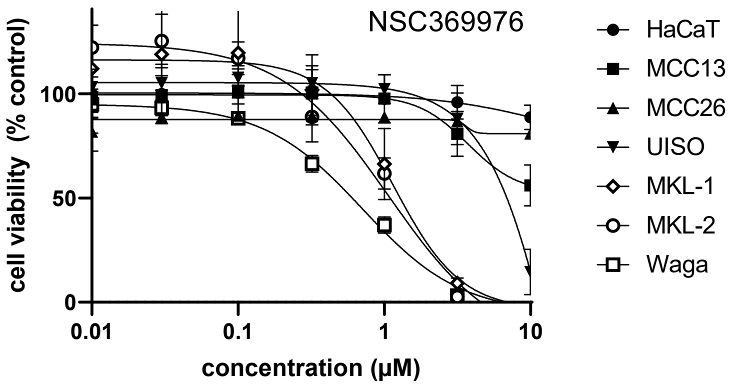

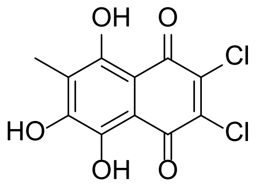

Cell line	µM IC_{50} (95% CI)
HaCaT	> 10
MCC13	~10
MCC26	> 10
UISO	~5
MKL-1	1.15 (0.68-2.71)
MKL-2	1.07 (0.72-2.02)
WaGa	0.71 (0.59-0.86)

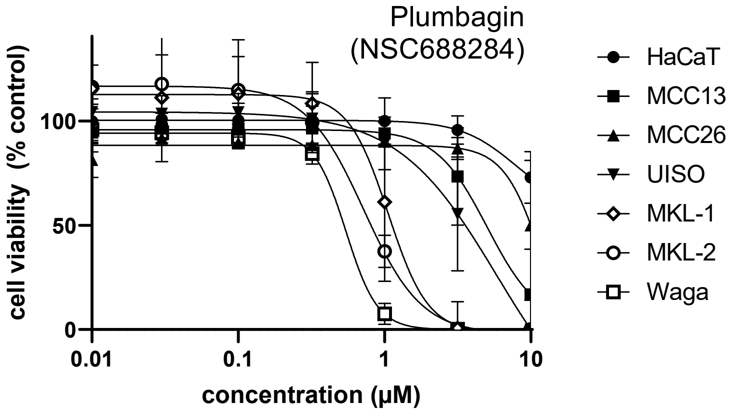

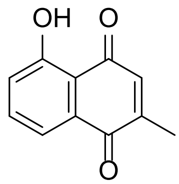

Cell line	µM IC_{50} (95% CI)
HaCaT	> 10
MCC13	~5
MCC26	~10
UISO	~6
MKL-1	1.06 (0.66-2.01)
MKL-2	0.73 (0.54-0.95)
WaGa	0.55 (0.48-0.61)

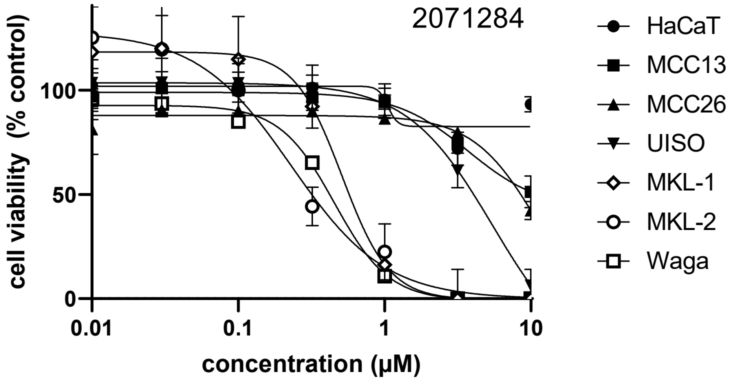

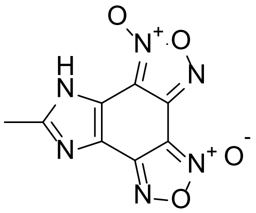

Cell line	µM IC_{50} (95% CI)
HaCaT	> 10
MCC13	> 10
MCC26	~10
UISO	5.5 (> 3.4)
MKL-1	0.51 (< 0.75)
MKL-2	0.23 (0.15-0.36)
WaGa	0.45 (0.41-0.51)

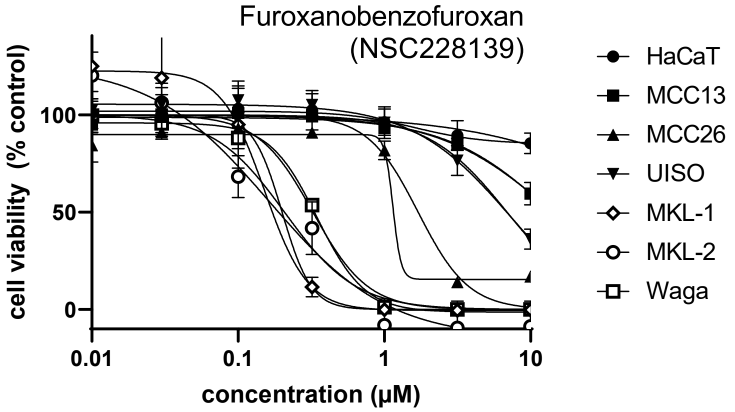

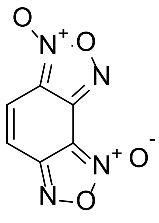

Cell line	µM IC_{50} (95% CI)
HaCaT	> 10
MCC13	> 10
MCC26	1.70 (1.35-2.15)
UISO	6.91 (5.75-8.40)
MKL-1	0.15 (< 0.22)
MKL-2	0.16 (0.07-0.28)
WaGa	0.35 (0.31-0.39)

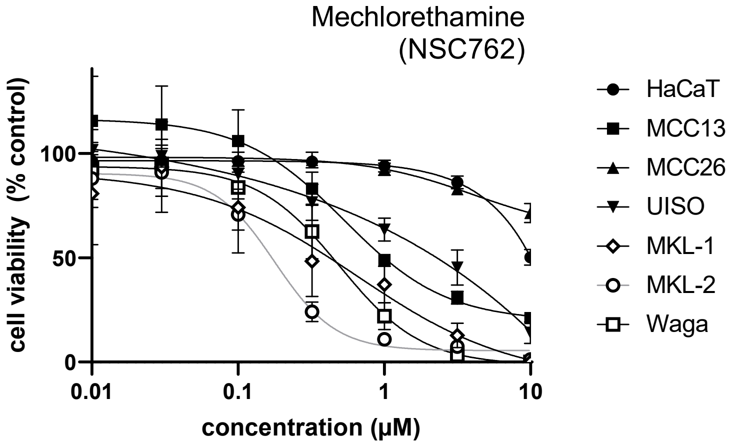

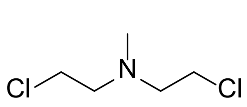

Cell line	µM IC$_{50}$ (95% CI)
HaCaT	~10
MCC13	0.53 (0.31-1.21)
MCC26	> 10
UISO	~5
MKL-1	~0.6
MKL-2	0.18 (0.13-0.25)
WaGa	0.49 (0.34-0.73)

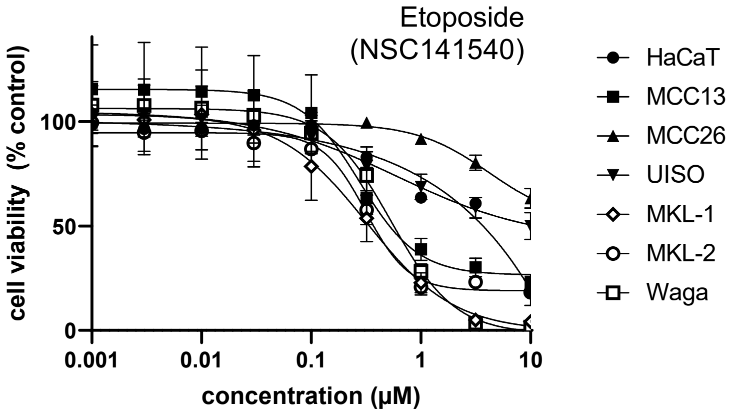

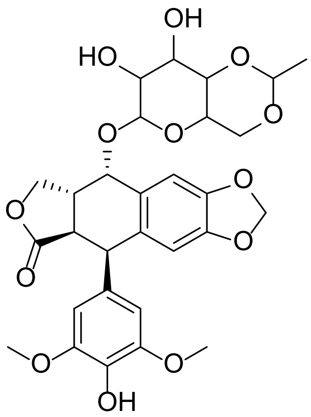

Cell line	µM IC$_{50}$ (95% CI)
HaCaT	~10
MCC13	0.28 (0.17-0.52)
MCC26	~10
UISO	~10
MKL-1	0.33 (0.19-0.64)
MKL-2	0.31 (0.24-0.40)
WaGa	0.54 (0.42-0.70)

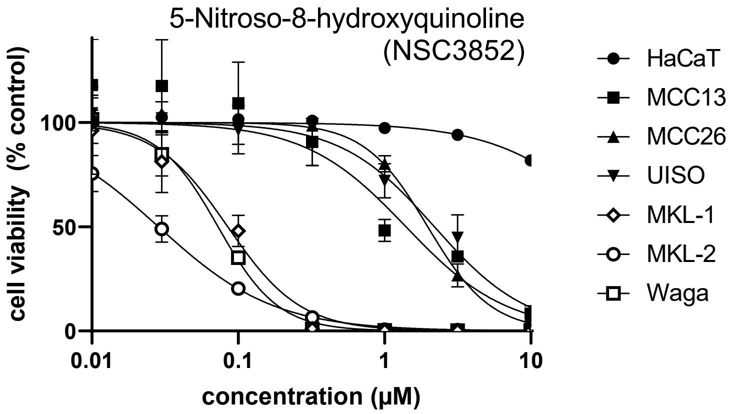

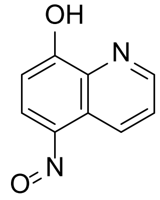

Cell line	µM IC$_{50}$ (95% CI)
HaCaT	> 10
MCC13	1.40 (0.83-2.46)
MCC26	1.97 (1.08-2.16)
UISO	2.91 (1.68-2.83)
MKL-1	0.08 (0.07-0.11)
MKL-2	0.03 (0.02-0.03)
WaGa	0.07 (0.06-0.08)

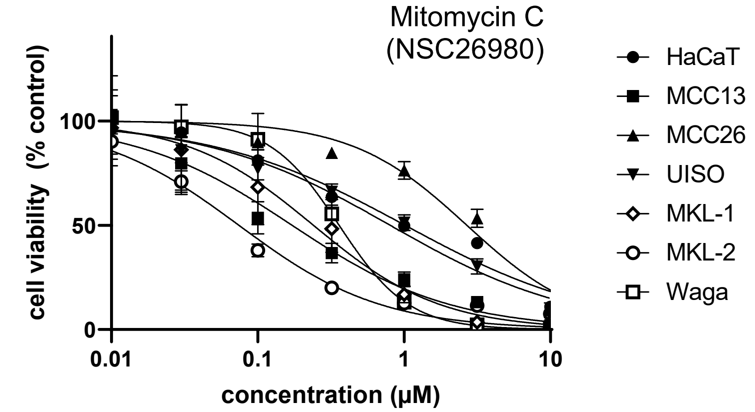

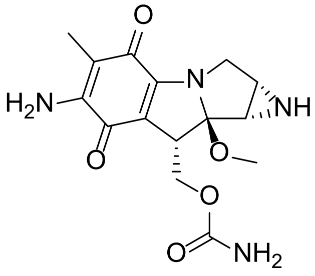

Cell line	µM IC_{50} (95% CI)
HaCaT	1.02 (0.82-1.27)
MCC13	0.17 (0.12-0.25)
MCC26	2.69 (2.14-3.35)
UISO	0.80 (0.64-1.02)
MKL-1	0.24 (0.16-0.35)
MKL-2	0.07 (0.06-0.09)
WaGa	0.36 (0.31-0.42)

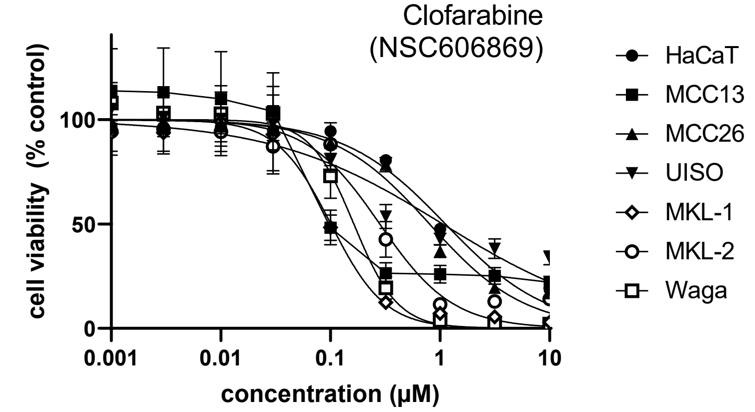

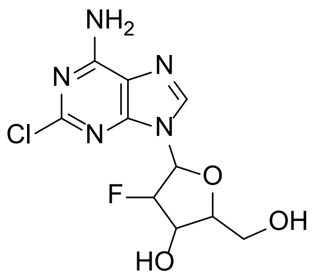

Cell line	nM IC_{50} (95% CI)
HaCaT	1122 (974-1295)
MCC13	184 (87-144)
MCC26	803 (673-962)
UISO	1059 (700-1663)
MKL-1	101 (81-126)
MKL-2	282 (211-379)
WaGa	164 (136-197)

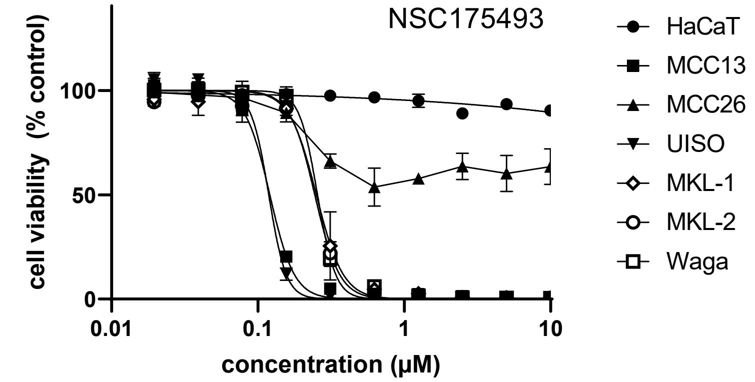

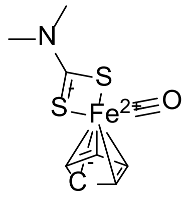

Cell line	µM IC_{50} (95% CI)
HaCaT	> 10
MCC13	0.12 (0.11-0.13)
MCC26	ND
UISO	0.12 (0.11-0.13)
MKL-1	0.25 (0.23-0.27)
MKL-2	0.24 (0.23-0.26)
WaGa	~0.25

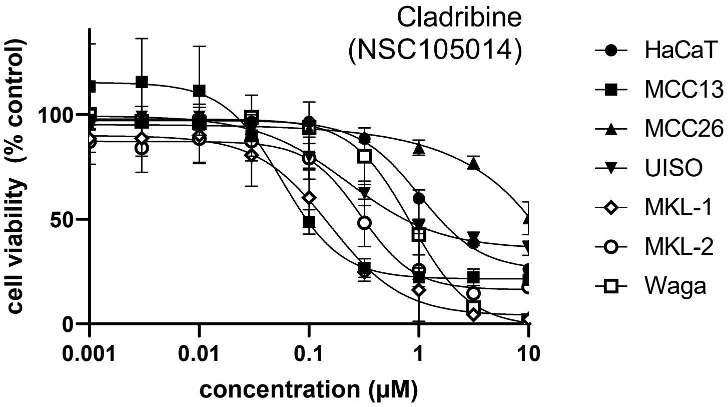

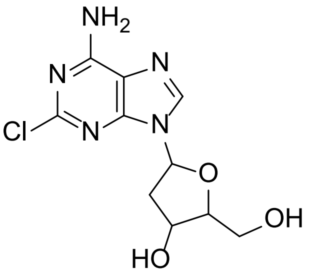

Cell line	nM IC_{50} (95% CI)
HaCaT	1015 (960-1154)
MCC13	56.9 (38.4-83.9)
MCC26	~10,000
UISO	227 (165-322)
MKL-1	157 (165-322)
MKL-2	290 (183-495)
WaGa	838 (595-1288)

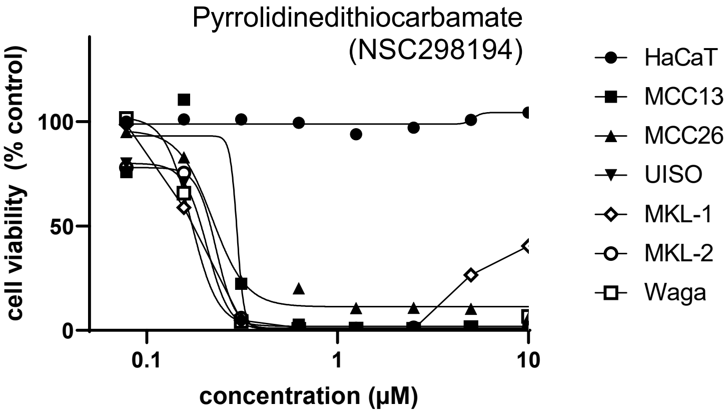

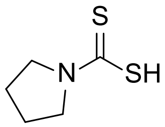

Cell line	µM IC_{50} (95% CI)
HaCaT	> 10
MCC13	~0.3
MCC26	~0.2
UISO	0.21 (0.20-0.24)
MKL-1	0.17 (0.16-0.17)
MKL-2	0.23 (0.22-0.24)
WaGa	0.17 (< 0.19)

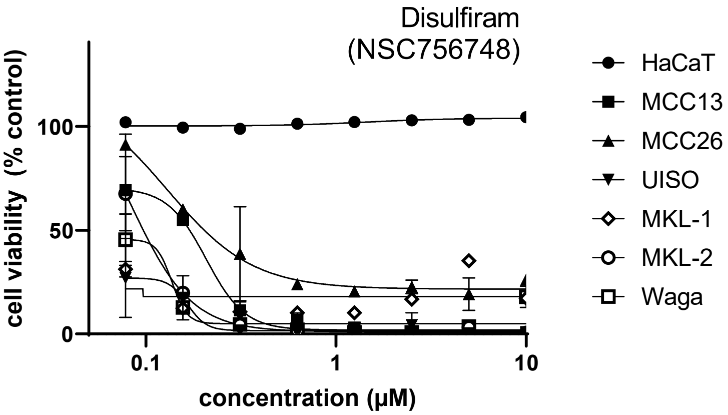

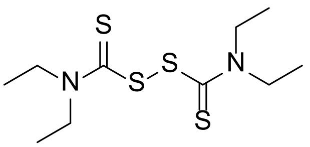

Cell line	µM IC_{50} (95% CI)
HaCaT	> 10
MCC13	0.14 (0.11-0.18)
MCC26	0.29 (0.08-0.70)
UISO	0.04 (0.02-0.06)
MKL-1	< 0.01
MKL-2	0.10 (0.09-0.10)
Waga	0.07 (0.03-0.09)

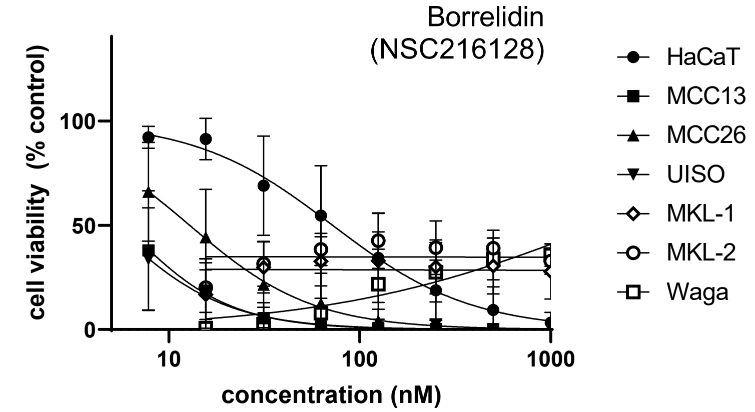

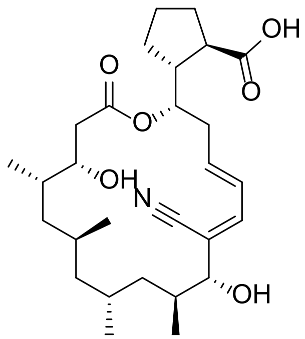

Cell line	nM IC_{50} (95% CI)
HaCaT	72 (53-99)
MCC13	5.8 (2.8-7.8)
MCC26	12.9 (8.9-17.1)
UISO	5.0 (1.9-7.1)
MKL-1	ND
MKL-2	ND
WaGa	ND

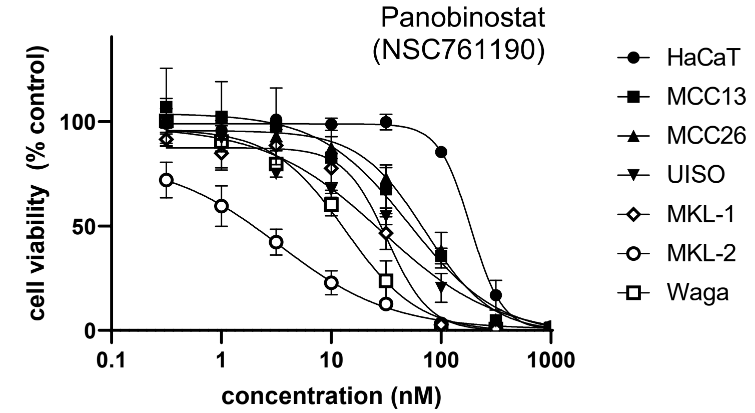

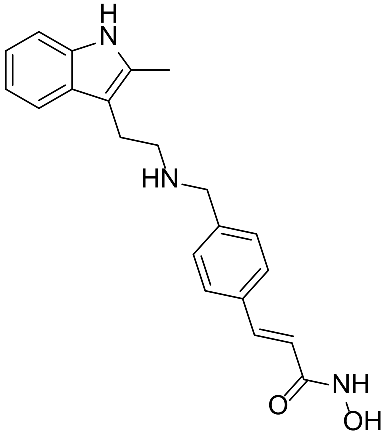

Cell line	nM IC_{50} (95% CI)
HaCaT	186 (171-201)
MCC13	51 (32-79)
MCC26	72 (62-83)
UISO	28 (18-41)
MKL-1	32 (28-37)
MKL-2	3.4 (1.5-5.2)
WaGa	14 (10-17)

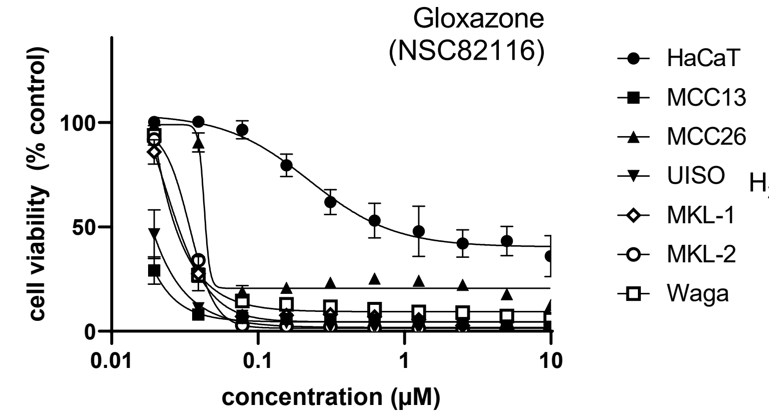

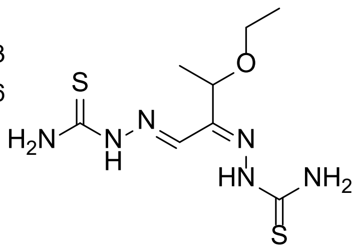

Cell line	µM IC_{50} (95% CI)
HaCaT	0.22 (0.15-0.31)
MCC13	< 0.02
MCC26	~0.04
UISO	< 0.02
MKL-1	~0.02
MKL-2	0.03 (0.03-0.04)
WaGa	~0.03

Natural product source organisms, active compound isolation and structure elucidation.

Specimens of the marine sponge *Acanthostrongylophora* sp. were collected in the Northern Territory, Australia in June 2003. A voucher specimen (voucher ID # 0M9H2507) was deposited at the Smithsonian Institution, Washington, D.C. The frozen sponge sample (274 g wet weight) was ground and processed using the standard NCI method for marine samples to provide 6.3 g of organic solvent (CH_2Cl_2-MeOH, 1:1) extract (NSC # C025917). (McCloud, T.G., *Molecules*. **15**, 4526-4563 (2010))

A 443 mg aliquot of the organic solvent extract was separated on diol solid phase extraction cartridges sequentially eluted with hexane/CH_2C_{12} (9:1), CH_2Cl_2/EtOAc (20:1), 100% EtOAc, EtOAc/ MeOH (5:1), and 100% MeOH. The 100% MeOH fraction was further purified on a Sephadex LH-20 column (2 x 92 cm) that was eluted with CH_2Cl_2/MeOH (1:1). Final purification was achieved by C_{18} HPLC using a Phenomenex Luna C18 column (10 x 250 mm) and eluting with a linear CH_3CN/H_2O gradient from 5:95 to 100% CH_3CN over 30 min at a 4 mL/min flow rate with 0.05% TFA (vol/vol) to provide 3 mg of petrosamine B. Petrosamine B was identified by comparing its NMR spectroscopic data and MS spectrometric data with literature values (Carroll AR, Ngo A, Quinn RJ, Redburn J, Hooper JNA. Petrosamine B, an inhibitor of the *Heliobacter pylori* enzyme aspartyl semialdehyde dehydrogenase from the Australian sponge Oceanapia sp. *J. Nat. Prod.* **68**, 804-806, 2005).

The *Clonostachys rosea* isolate (MI4762 TV8-1) was obtained from a soil sample collected in Macomb, Michigan, USA, which had been submitted to the Citizen Science Soil Collection Program at the University of Oklahoma. The *Penicillium* sp. isolate (23 BIA-6) was obtained from sediment collected from Lake Michigan, USA (43.116667, -86.533333 GPS coordinates). Cryogenically preserved samples of both fungi have been archived in the University of Oklahoma Fungal Repository. For identification of the isolates, the nucleotides spanning the ITS1-5.8S-ITS2 regions of genomic DNA were sequenced. The resulting sequences were compared to data available through GenBank. Matches at the 100% identity levels were found for both isolates. The fungus from soil sample MI4762 TV8-1 was identified as *Clonostachys rosea*, while the isolate from 23 BIA-6 was identified as a *Penicillium* sp. Sequence data for both isolates have been deposited in Genbank (*C. rosea* GenBank accession number MW466525, and *Pencillium* sp. GenBank accession number MW714610). For studies of the natural products generated by the isolates, the fungi were grown on Cheerios® breakfast cereal supplemented with 0.3% sucrose and 0.005% chloramphenicol in three large mycobags (Unicorn Bags, Plano, TX, USA). Fungal biomass from both cultures were extracted overnight in ethyl acetate. The resulting organic extracts were subjected twice to partitioning against water (1:1, vol:vol). The ethyl acetate layers were retained, and the solvent was evaporated *in vacuo*, yielding 18 g of deep red colored material from the *C. rosea* culture and 56 g of brown-red colored material from the *Pencillium* sp. culture.

The fungal extract of *Clonostachys rosea* (OK1_MI4762 TV8-1) was subjected to diol column chromatography using a step gradient elution with 100% hexane, CH_2Cl_2, EtOAc, acetone, and MeOH. The fraction that eluted with CH_2Cl_2 was purified by preparative reversed-phase HPLC (Varian Dynamax C18 column, 20 mm × 250 mm; 9.0 mL/min, CH_3CN-H_2O gradient (20:80-100:0)), yielding 13 peaks rich in secondary metabolites. Purification of the sub-fraction (8) was accomplished by analytical HPLC (Phenomenex Luna C18 column, 4.6 mm × 250 mm; 0.9 mL/min, CH_3CN-H_2O gradient (50:50-100:0)), to yield asperphenamate (1.5 mg). Asperphenamate was identified by analysis of its spectroscopic (NMR) and spectrometric (ESI-MS) data and through comparisons with literature data (Songue JL, Kouam, Dongo E, Mpondo TN, White RL. Chemical constituents from stem bark and roots of *Clausena anisata*. *Molecules*, **17**, 13673-13686, 2012).

The fungal extract of (OK1_23 BIA-6) was subjected to diol column chromatography using a similar gradient elution system with 100% hexane, CH_2Cl_2, EtOAc, acetone, and MeOH. The fraction that eluted with EtOAc was separated by preparative reversed-phase HPLC (Varian Dynamax C18 column, 20 mm ×

250 mm; 9.0 mL/min, CH_3CN-H_2O gradient (20:80-100:0)) to yield mycophenolic acid (4.2 mg). Mycophenolic acid was identified by analysis of its spectroscopic (NMR) and spectrometric (ESI-MS) data and through comparisons with literature data (Danheiser RL, Gee SK, Perez JJ. Total synthesis of mycophenolic acid. *J. Am. Chem. Soc.* **108**, 806-810, 1986).
